# What Do Microglia Really Do in Healthy Adult Brain?

**DOI:** 10.3390/cells8101293

**Published:** 2019-10-22

**Authors:** Marcus Augusto-Oliveira, Gabriela P. Arrifano, Amanda Lopes-Araújo, Leticia Santos-Sacramento, Priscila Y. Takeda, Daniel C. Anthony, João O. Malva, Maria Elena Crespo-Lopez

**Affiliations:** 1 Laboratório de Farmacologia Molecular (Instituto de Ciências Biológicas), Universidade Federal do Pará, Belém 66075-110, Brasil; marcusoliveira@globo.com (M.A.-O.); gabrielaarrifano@uol.com.br (G.P.A.); amanda.lopes1647@gmail.com (A.L.-A.); yukitakeda98@gmail.com (P.Y.T.); 2 Laboratory of Experimental Neuropathology, Department of Pharmacology, University of Oxford, Oxford OX1 3QT, UK; daniel.anthony@pharm.ox.ac.uk; 3 Faculty of Medicine, Coimbra Institute for Clinical and Biomedical Research (iCBR), and CNC.IBILI, University of Coimbra, Coimbra 3000-548, Portugal; jomalva@fmed.uc.pt

**Keywords:** cognition, adult neurogenesis, plasticity, memory, CNS, healthy, glia, homeostasis, learning, synapse

## Abstract

Microglia originate from yolk sac-primitive macrophages and auto-proliferate into adulthood without replacement by bone marrow-derived circulating cells. In inflammation, stroke, aging, or infection, microglia have been shown to contribute to brain pathology in both deleterious and beneficial ways, which have been studied extensively. However, less is known about their role in the healthy adult brain. Astrocytes and oligodendrocytes are widely accepted to strongly contribute to the maintenance of brain homeostasis and to modulate neuronal function. On the other hand, contribution of microglia to cognition and behavior is only beginning to be understood. The ability to probe their function has become possible using microglial depletion assays and conditional mutants. Studies have shown that the absence of microglia results in cognitive and learning deficits in rodents during development, but this effect is less pronounced in adults. However, evidence suggests that microglia play a role in cognition and learning in adulthood and, at a cellular level, may modulate adult neurogenesis. This review presents the case for repositioning microglia as key contributors to the maintenance of homeostasis and cognitive processes in the healthy adult brain, in addition to their classical role as sentinels coordinating the neuroinflammatory response to tissue damage and disease.

## 1. Introduction

Microglial cells are the most prominent immune cells of the central nervous system (CNS) and are the first to respond when something goes wrong in the brain [1]. The microglial population accounts for approximately 10% of the cells in the whole brain [2]. Over the years, the immune functions of these cells have attracted attention due to their involvement in virtually all pathological processes in the brain, including inflammation, stroke, neurodegenerative diseases, and viral and bacterial infection [3,4]. However, their role in supporting cognitive processes and homeostasis in the healthy adult brain is only beginning to be understood, with recent studies generating striking new information for the field.

In this review, we explore the neurobiology of microglia in the healthy adult brain and discuss the evidence for microglia influencing the cognitive processes of learning and memory, directly or indirectly, as well as their contribution to the homeostatic maintenance of the brain (Table 1).

## 2. Overview of the Neurobiology of Microglia

In the late 1990s, microglia were shown to arise from yolk sac-primitive macrophages and proliferate in situ during the embryogenic period [38]. This finding was only confirmed in 2010 by Ginhoux and colleagues using fate mapping analysis [39], breaking the dogma that microglia are derived from peripheral macrophages.

The first microglia can be detected in the brain by immunohistochemistry as early as E9.5 [39,40]. This microglial invasion is thought to occur in two waves, one on E9.5 and another on E14.5, at which point the final progenitor population is established and after which the number of microglia increases gradually [38,41,42]. Approximately 95% of the microglial population is formed in the first 2 postnatal weeks [38]. In humans, microglia enter the brain early before other critical events, such as peaks of neurogenesis, astrogliogenesis, oligodendrogenesis, myelination, and the emergence of vasculature and the blood-brain barrier between gestational weeks 4 and 24 [43].

Once established, the microglial population is maintained by local proliferation, without significant participation of hematopoietic progenitors [39]. Reinforcing the absence of perinatal monocyte infiltration in the formation of the microglial population, it was recently demonstrated that microglial renewal occurs via a coupled process of proliferation and apoptosis over the lifespan of the organism in both mice and humans [44].

The microglial population is not uniformly distributed throughout the adult brain and can vary in density up to 5-fold in different brain regions [45]. In addition, there are temporal and spatial differences in function and transcriptional profile [46,47,48], which appear to be regulated by different neural circuits and age [49]. According to data from transcriptional and epigenomic studies, microglia can be classified into three stages with distinct gene expression and functional profiles: early, preadult, and adult [49].

Classically, microglia are known as the major players in brain defense, changing their morphology in response to changes in the local environment. Accordingly, these cells are often classified in a system derived from the classification of peripheral macrophages as M1 (pro-inflammatory profile) and M2 (anti-inflammatory profile), which oversimplifies the diverse complexity of microglia [50]. A more useful classification system is needed to incorporate the transcriptomic profiles, regional and temporal heterogeneity, and observed patterns of response in microglia [51]. For example, microglia respond differently to diverse pathological stimuli and change their behavior over the evolution of any given pathology [52] in relation to different phagocytic activities [53] and transcriptional profiles [47,54] in different brain regions and at different ages. Notably, polarization into the “classically activated” M1 phenotype can be blocked in microglia after spinal cord injury by deletion of TMEM16F, a protein that functions as a Ca^2+^-dependent ion channel and phospholipid scramblase [55], which provides a new tool to study microglial function. This protein has also been shown to be essential for the development of neuropathic pain in mice [56]. Microglia have long been recognized as key contributors to neuropathic pain in which the upregulation of microglial purinergic P2X4 receptors and increased release of brain-derived neurotrophic factor (BDNF) seem to be essential contributors [57]. BDNF-TrKB signaling alters KCC2 function, leading to a reduced chloride extrusion capacity and dampened GABAAR/GlyR-mediated inhibition.

Microglial cells maintain the neural environment under constant vigilance [5,6], monitoring the functional states of synapses [7]. These cells are decorated by a wide range of receptors, including fractalkine receptors and colony-stimulating factor 1 receptors (CSF1Rs), making them highly sensitive to any changes in cerebral homeostasis [11]. For example, the stimulation of CSF1R by CSF1 and interleukin (IL)-34 can induce expansion of CD11c+ microglia, resulting in the amelioration of experimental allergic encephalomyelitis (EAE) symptoms and decreased demyelination [58]. Thus, to prevent immune activation and maintain surveillance and homeostatic functions, microglia appear to be maintained in a ‘non-inflammatory’ state. Although this system may promote the protective roles played by microglia in the CNS, the breakdown of this system by disease or aging leads to an impaired microglial neuroprotective capacity and increased neuroinflammation [59]. For example, the behavior of microglia is held in check by CD200L-CD200R interactions, and CD200 has been demonstrated to be reduced in the brains of individuals with Alzheimer’s disease (AD). Specific upregulation of neuronal CD200 was reported to promote cognitive function in an AD model by inhibiting microglial activation and secretion, improving synaptic function, and preventing synaptic loss [60]. In a study of individuals living with HIV on effective antiretroviral therapy, when microglial activation was probed with PET-based translocator protein 18 kDa (TSPO) ligands, cognition was inversely associated with microglial activation [61].

Microglia respond to tissue injury or disease through receptors for neurotransmitters and receptors that recognize danger-associated molecular patterns (DAMPs), such as adenosine triphosphate (ATP), and pathogen-associated molecular patterns (PAMPs), such as lipopolysaccharide (LPS) and viral DNA and RNA. These receptors provide signals that regulate the change in microglial profile from homeostatic to active states [4]. Furthermore, a subgroup of microglia has been found in areas of neurodegeneration, so-called disease-associated microglia, that detect damage in the form of neurodegeneration-associated molecular patterns (signals released upon neurodegenerative processes) and play positive and negative roles [59,62].

Despite their well-recognized role in neuroprotection, the global absence of microglia does not appear to cause overt cognitive or behavioral deficits in adult mice [63]. These surprising findings have been achieved through the use of new pharmacological interventions such as selective kinase inhibitors of CSF1R (such as PLX3397, PLX647, Ki20227, GW2580) and desatinib, a nonselective kinase inhibitor which can generate ~99% depletion. In the experiments with these compounds, the control and experimental groups showed no difference in behavioral tests (open field, elevated plus maze, Barnes maze, and contextual fear conditioning). These results support the conclusion that microglia do not play any roles in cognitive functions in the normal adult brain and were surprising to most glial cell biologists [63]. However, other recent findings indicate that microglia contribute to normal behavior, and it is possible that the tests in mice are not sensitive to subtle changes in behavior. For example, Torres and colleagues showed that local elimination of hippocampal microglia using clodronate as a microglial toxin impaired the performance of mice in spatial memory tests. These impairments recovered after microglial repopulation [26]. However, though microglial elimination transiently altered spatial memory, it did not affect social behavior [26]. It is hard to reconcile the outcomes of these apparently contradictory conclusions in the literature. However, an understanding of the basal contribution of microglia is essential if we are to understand the implications of manipulating microglial populations. As a consequence of the seeming lack of impact of CSFR1 inhibitor on brain function, these compounds are being hailed as potential therapies for neurodegenerative disease and acute injuries, such as stroke.

## 3. Role of Microglia in Homeostatic Functions and Cognitive Processes

Among the roles assigned to glial cells in the healthy adult brain, astrocytes affect the cognitive processes of learning and memory, whereas microglia are classically thought to be immune elements. Astrocytes are intensely involved with cognition-related processes, such as the regulation of synaptic activity [64], adult neurogenesis [65], energetic supply in neuronal support [66], and the maintenance of homeostasis [67]. Oligodendrocytes also play a key role in cognitive processes [68]. For example, the production of new oligodendrocytes was recently demonstrated to be required for motor-skill learning [69], and that the motor learning promotes myelin plasticity in humans, reflecting learning-related increases in myelination [70,71,72]. Thus, while the focus has been on astrocytes and oligodendrocyte for the maintenance and cognitive function there is evidence that microglia do interact with synaptic and cognitive processes.

Using real-time in vivo microscopy, microglia were shown to exhibit highly dynamic changes in their cellular morphology, highlighting that microglia monitor and respond to the functional status of synapses [7]. Indeed, the approximate velocity of the processes of resting microglia was found to be about 1.47 μm/min, and this motility is altered by the increased synaptic and neuronal activity [5]. These structural interactions were shown to be activity-dependent because contact frequency can be reduced by decreased neuronal activity following either binocular eye enucleation, injection of tetrodotoxin into both retinae, or a reduction of body temperature [7]. Furthermore, glutamate release induces microglial process extension in CA1 in hippocampal slices. Under pathologic conditions, microglial cells increase the expression of the glutamate transporter GLT-1 (EAAT-2), which may play a role in glutamate clearance [73]. The induced expression of glutamate transporter suggests a neuroprotective role of microglia against glutamate excitotoxicity following nerve axotomy [74]. Thus, we might speculate that microglia contribute to glutamate clearance in the healthy brain in regions where neuronal activity is markedly increased.

However, Fontainhas has argued that the motility control of microglial processes is mediated through a purinergic signaling [13] as it was evident that lowering extracellular ATP concentration by the ATP-hydrolyzing enzyme apyrase results in reduced process movements; conversely, artificially created ATP gradients stimulate their motility [6]. The metabotropic P2Y12 purinoceptors have been shown to be responsible for this behavior [75]. Using in vivo two photon imaging, Akiyoshi and colleagues have recently demonstrated that synaptic activity is increased after microglial processes contact spines, which is not detected when microglia are activated by LPS [28] and may explain how an immune challenge in the brain might lead to altered neuronal function. In addition to the brief contacts with pre- and postsynaptic terminals [7], microglial processes are also found in contact to the peri-synaptic astrocytic processes and synaptic clefts [8]. Together, these findings show that microglia can directly influence the synapse activity in the healthy adult brain and lead to the synchronization of local populations of neurons [28].

During development, the maturation of neuronal circuits requires selective elimination of synaptic connections. Neuron-intrinsic mechanisms are important in this process, but microglia also play a critical role in this clearance where they survey and then selectively phagocytose pre- and postsynaptic elements in a neuronal activity-dependent manner in the healthy developing brain. This process was first recognized in the facial nerve injury model and termed synaptic stripping [76], but only many years later direct evidence was produced to show that the ‘synaptic pruning’ by microglia is necessary for normal brain development [77]. During postnatal development, Paolicelli and colleagues demonstrated the synaptic material engulfment by microglia [77]. They found specific proteins from presynaptic (SNAP25) and postsynaptic (PSD95) elements within microglial processes after synaptic contact, suggesting that microglial synaptic pruning is critical for normal brain development. Indeed, microglial dysfunction [78] or depletion [79] during neurodevelopment is known to affect synaptogenesis and synaptic pruning, and give rise to cognitive dysfunctions [80].

Microglia have also been reported to contribute to the selective elimination of synapses in an indirect manner. For example, refinement of the climbing fiber to Purkinje cell innervation is severely impaired in the absence of microglia, but in this case there is no morphological evidence suggesting engulfment of climbing fibers by microglia [81]. Whatever the mechanism, the number, morphology, and function of synaptic contacts in the developing brain is profoundly altered in the absence of microglia. However, there is accumulating evidence that synaptic pruning continues into adolescence and adulthood. For example, in the rat there is a significant increase in microglial engulfment of spines at post-natal day 39 relative to earlier ages, which then declines by day 50 [82].

It has been suggested that aberrant synaptic stripping at this age might led to schizophrenia. In adulthood, microglial synapse refinement may contribute to the elimination of weak synapses during the sleep phase [83]. Activity-dependent down-selection of synapses is held to explain the benefits of sleep on memory acquisition, consolidation, and integration during the day and microglia seem to have a role in this process. Immunohistochemistry and immunoblotting revealed the presence of synapses and synaptic proteins in microglia at increased levels at zeitgeber time (ZT) 0 compared with ZT 12 in two-month old rats [83]. However, others have shown that while there is an increased density of excitatory synapses in the granule cells of the dentate gyrus of sleep deprived adolescent mice (5 weeks old), this is not true of adult mice [84]. A full analysis of the circadian cycle was not performed in this study and thus this finding does not preclude the involvement of microglia in the renormalization of net synaptic strength and restoration of cellular homeostasis during sleep. Interestingly, microglia are also known to exhibit phagocytic behavior in aged animals where deficits in auditory function are associated with microglia that have been shown to contain axon-like or dendritic spine inclusions [27]. Together, these data suggest a continuum in the synaptic pruning function in the adult brain that is related to synaptic activity.

Microglia express a wide range of receptors for a large number of neurotransmitters, such as glutamate, GABA, norepinephrine, cannabinoids, acetylcholine, and ATP [9,10,11]. Oddly, macrophages have been shown to contain high concentrations of GABA [85]. The presence of receptors for the classical neurotransmitters allows these cells to respond to synaptic activity by releasing immunotransmitters that are known to exert strong effects on neurons, such as chemokines and cytokines. Many neuronal populations are decorated with receptors, such as those for tumor necrosis factor-α (TNF-α) and interferon-γ (IFNγ) [9,12]. While cytokine expression in the brain is usually considered pathological, it has become clear that low levels of expression may mediate neuron-microglia crosstalk in the uninjured adult brain. For example, CX3CL1 [86] and complement receptors [87] are known to mediate synaptic pruning, and TNF-α at picomolar concentrations, which is only produced by microglia in the normal brain, is known to modulate synaptic scaling [88]. This is the uniform adjustment in the strength of all synapses formed on a given neuron in response to prolonged changes in the electrical activity. Interestingly, the scaling effect of TNF-α seems to be mediated indirectly by the activation of tumor necrosis factor receptor 1 (TNFR1) on astrocytes, leading to the release of astroglial ATP and glutamate, which then affects synaptic transmission through presynaptic metabotropic glutamate receptors [89].

In support of the neuromodulatory role of microglial derived TNF-α, it has recently been shown that the long-term inhibition of TNF-α with the nonselective TNF-α inhibitor etanercept impairs learning and memory after two months of treatment [90]. It also decreased adult neurogenesis (see below). The interleukin-1 (IL-1) cytokine family (IL-1α, IL-1β, and the IL-1 receptor antagonist), which is also involved in immune and inflammatory responses both in the brain and in the periphery has also been shown to have a role in learning and memory in the normal brain. In particular, IL-1α activity in the CNS appears to be important for hippocampal memory processing [91]. Indeed, young IL-1r1 knockout mice but not IL-1β knockout mice showed an impairment in long-term memory extinction, which also suggests that IL-1α might facilitate memory extinction [92]. However, while microglia make and release IL-1 α/β [12], it is clear that neurons can also make IL-1 and thus it is harder to ascribe a distinct role for microglial-derived IL-1 in neuromodulation in the normal brain.

*In vitro* and *in vivo* experiments have shown that, in addition to the release of immunotransmitters, microglial morphology is controlled by glutamatergic and GABAergic neurotransmission [13,14]. However, though microglia have been suggested to express functional N-methyl-D-aspartate (NMDA) receptors [93], microglia lack electrical responses to localized puffs of glutamate or NMDA [94]. Thus, microglial process extension is most likely mediated by neuronal, but not microglial, NMDA receptors. For example, in the visual cortex, almost all microglial processes contact synapses at given any particular moment, and a reduction in the visual stimulus, decreasing the synaptic input to the visual cortex, decreases the motility of microglial processes. This phenomenon is reverted by visual re-stimulation, suggesting that microglial surveillance behavior is related to neuronal activity [8]. However, precisely what the microglial processes are doing in these circumstances remains unclear.

**Figure 1:**
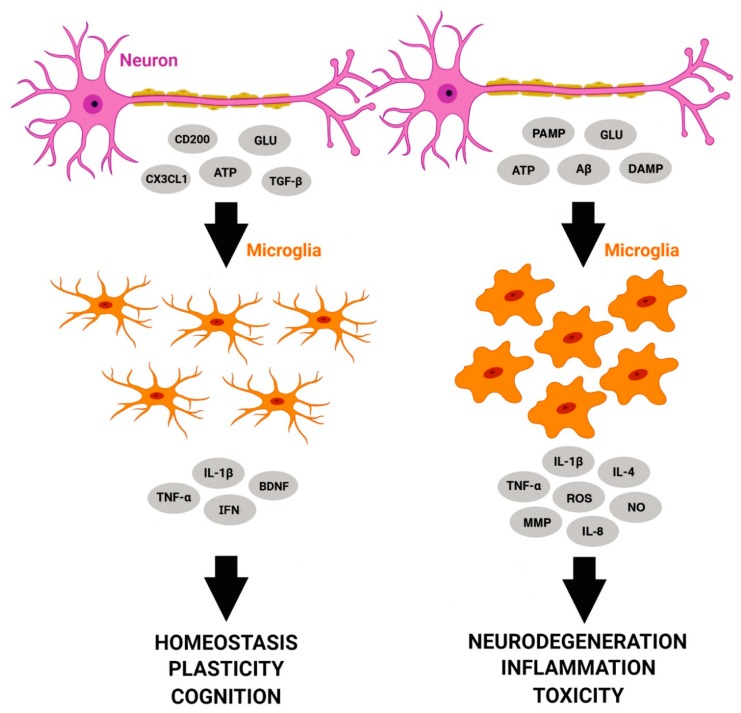
**Microglial responses to different molecules released by neurons**. CX3CL1, CD200, glutamate, ATP, and TGF-β induce an anti-inflammatory microglial profile, with microglia performing housekeeping tasks and contributing to homeostasis, plasticity, and cognition processes through release of TNF-α, IL-1β, IFN, and BDNF. In a different scenario, ATP, glutamate, danger-associated molecular patterns (DAMPs), pathogen-associated molecular patterns (PAMPs), and amyloid-beta proteins drive microglial behavior to a more responsive pro-inflammatory state, releasing IL-1β, TNF-α, IFN-γ, IL-4, ROS, NO, IL-8, and MMP. When the pro-inflammatory profile is maintained for a long time, it foments pathological conditions, such as toxicity, neuroinflammation, and neurodegeneration.

Certain molecules such as purines, cytokines and glutamate act as ‘On’ signals, inducing pro-inflammatory or neuroprotective microglial profiles [17]. Purines, such as ATP, as described above, show the ability to mediate microglial migration to sites of damage [6]. In such cases, an increased level of purines such as uridine triphosphate (UTP) and ATP in the extracellular space may promote microglial migration, wrapping, and stripping activities [15,16]. Other molecules such as CD200 or fractalkine/CX3CL1 act as ‘Off’ signals, and are reported to oppose such actions of ATP [95,96,97] and keep microglia in a housekeeping mode that contributes to neuronal plasticity, and antagonizes the development of the pro-inflammatory phenotype [17] (Figure 1). TREM2 also plays a critical function in inducing the “Off” phenotype in microglia, as the lack of this receptor leads to inhibition of phagocytosis of apoptotic neurons and increased gene transcription of pro-inflammatory molecules such as TNF-α and NO synthase-2 [22]. It is important to note that the clearance activity performed by microglia through TREM2 occurs in the absence of inflammation.

The imbalance of this neuron–glia communication ‘On’ vs. ‘Off’ may disrupt the homeostasis, causing synaptic deficits and eventually leading to cognitive impairment. Reduced CD200 expression, with consequential down-regulation of the “Off” system, leads to microglial activation, impairing synaptic plasticity [18]. CD200^−/−^ mice exhibit long-term potentiation (LTP) deficits and impaired synaptic plasticity [19]. CD200 fusion protein, which can activate CD200R, decreases microglial activation and LTP deficits in aged rats [98].

Also, CX3CL1 signaling has been reported to contribute to the maintenance of brain homeostasis [20]. CX3CL1 is able to depress synaptic transmission in the hippocampus of rats, acting as a potent neuromodulator [21]. Mice lacking the CX3CR1 receptor present with contextual fear conditioning and Morris water maze deficits, with CX3CR1 deficiency affecting motor learning [99]. In addition, CX3CL1 upregulation in the rat hippocampus immediately after a spatial learning task suggests a role in synaptic plasticity [100]. This occurs through modulation of glutamate-mediated calcium levels, suggesting the modulation of synaptic scaling through glutamatergic transmission [100].

Other molecules, such as transforming growth factor beta-1 (TGFβ1), are implicated in microglial signaling and reinforce the importance of microglial cells in brain homeostasis. Recently, silencing TGFβ1 signaling in microglia was shown to disturb homeostasis [23]. The deletion of TGFβ receptor type II (TGFβr2) causes microglial activation and upregulation of priming markers, suggesting impairment of microglial quiescence and brain homeostasis, with upregulation of chemokines CXCL10 and CCL2 leading to increased immune cell signaling [23]. Furthermore, microglia play a crucial role in learning and memory in adult mice through BDNF signaling [24], with the removal of BDNF from microglia resulting in a loss of synaptic plasticity related to learning and memory via the tropomyosin-related kinase receptor B signaling pathway [24].

Surprisingly, though inhibitors of CSF1R have suggested that microglia play a marginal role in cognition, the repopulation of microglia in aged animals [25] has been shown to improve the spatial memory as assessed by the Morris water maze test [25]. At the molecular level, the repopulation restores neuronal gene expression that is changed by aging and reverses age-related hippocampal neuronal complexity and age-related deficits in LTP [25]. These findings suggest that microglial cells affect neurons by influencing their gene expression and strongly support brain homeostasis and cognitive processes.

In addition to the influence of microglia on synaptic activity by direct contact or the release of cytokines or neurotransmitters, other types of cell communication have been shown for the influence of microglia on synaptic activity in the healthy adult brain, including extracellular vesicles (EVs) [29,101]. EVs, including cell-released exosomes and microvesicles, contain protein, lipids, DNA, and RNA from the original cell and are involved in the transfer of their contents from one cell to another, such as neurons and glia [101]. Microvesicles released from microglia affect excitatory neurotransmission by stimulating the neuronal production of ceramide and sphingosine, both in vitro and in vivo [29]. Sphingosine facilitates transmitter release of synaptic terminals via facilitation of soluble N-ethylmaleimide-sensitive factor activating protein receptor (SNARE) formation, increasing excitatory transmission and modulating synaptic plasticity [29].

## 4. Role of Microglia in Adult Neurogenesis

In addition to critical roles in plasticity, regulating synaptic formation, strength, and relocation in the working brain, microglia have also been shown to play a role in a more drastic form of structural plasticity, the introduction of new neurons throughout life, with strong implications for cognitive processes [102]. Adult neurogenesis is a complex phenomenon occurring in virtually all taxonomic groups, though striking controversies exist about its function and occurrence in humans [103]. The addition of new neurons to the circuits is a long and complex process that involves the generation, migration, differentiation, and maturation of these cells, and only a few survive and integrate the neural circuitry [104].

In the last few years, evidence has accumulated to support a functional role of adult neurogenesis in the many aspects of brain function, but mainly in cognitive and memory processes [105]. In rodents, adult neurogenesis is up-regulated by exercise [106] and environmental enrichment [107] and down-regulated by aging and disease [108,109]. It is important to note that the conditions that affect adult neurogenesis also move cognitive processes in the same direction. Exercise and an enriched environment are critical to cognition, whereas aging and disease are detrimental [103]. Thus, it seems reasonable to conclude that the generation and integration of new neurons is essential for the maintenance of homeostasis and cognitive processes.

The generation of new neurons is intimately related to the normal process of learning and forgetting. New neurons appear to modulate the capacity of the hippocampus to acquire new memories, as well as the forgetting process through competition with older synapses [110]. Among all of the glial cells, more is known about the role of astrocytes in adult neurogenesis. Astrocyte-like cells generate new neurons with homeostatic conditions [111]. More recently, astrocytes were shown to integrate these new-born cells into the synaptic circuit by controlling dendritic maturation and the survival of the new neurons [112]. However, microglia also play important roles in adult neurogenesis.

The involvement of activated microglia in adult neurogenesis was first suggested about 20 years ago [113,114]. These first studies demonstrated that neuroinflammation is sufficient to inhibit adult neurogenesis, and anti-inflammatory treatment is able to restore it. These findings suggest that the impairment of adult neurogenesis by activated microglia may be associated with cognitive deficits in disease and aging [113,114]. Moreover, even prenatal inflammation by gestational treatment with LPS is able to impair hippocampal neurogenesis and recognition memory in adult offspring [115]. The overexpression of TGFβ1 in the dentate gyrus restored adult neurogenesis and related behavior [115]. However, the story is complicated by other studies in which microglial ‘activation’ was shown to lead to an increase in adult neurogenesis in response to an enriched environment [116]. In this case, suppression of the immune system decreased the generation of new neurons, even in an enriched environment [116]. Although the differences in the methods used by all of these studies and the different aspects of the brain environment can be partially responsible for the apparent discrepancies in these findings, the evidence demonstrates that microglia may not be pro- or anti-neurogenic per se, and their role would depend on the balance between the various molecules secreted by these cells in different activation scenarios, leading them to act in one direction or the other [117]. It also seems clear that much of the seeming contradiction arises from the lack of agreement over what is considered an ‘activated’ microglial cell. Diverse stimuli often generate microglia with similar morphological phenotypes but very different molecular profiles [118]. Therefore, closer examination seems to support the view that a more aggressive inflammatory response by microglia does not encourage neurogenesis and supports the use of agents that suppress the more M1-like phenotype.

Interestingly, direct microglial contact may not be essential to influence neurogenesis, and soluble factors released by microglia may be sufficient. Investigating the role of microglia in adult neurogenesis *in vitro*, Walton and colleagues showed that microglial depletion correlates with the loss of inducible neurogenesis, and that the microglial environment can rescue it [31]. The use of conditioned medium from regions containing microglia was sufficient to restore neurogenesis, indicating the importance of soluble factors released by microglia [31]. Accordingly, cell culture media from BV2 immortalized microglia was able to increase the proliferation of adult mouse-derived neural stem/progenitor cells compared to the same cells incubated with basic fibroblast growth factor and epidermal growth factor cell media [32].

In the healthy adult mouse brain, newborn neurons pass through a critical period, with many of them undergoing apoptosis in the early days [33]. In this environment, unchallenged microglial cells (with ramified morphology and low levels of CD11 and CD68 expression) quickly phagocytose and clear these apoptotic neurons, maintaining the homeostasis of the neurogenic cascade [33]. They appear to do this without the production of proinflammatory mediators. In agreement, ablation of microglia from the dentate gyrus is sufficient to inhibit hippocampal adult neurogenesis [34]. Microglial depletion does not disturb neuroblast differentiation or maturation, but it does impair dentate gyrus neuroblast survival [34]. Interestingly, microglial cells from the dentate gyrus present a unique profile of RNA expression and respond exclusively to neurogenic factor VEGF compared to microglia from elsewhere in the brain, which suggests that microglia from the neurogenic zone are programmed to support adult neurogenesis in a way that other populations are not [34]. Recent studies have demonstrated that some receptors are able to modulate the influence of microglia on adult neurogenesis. For example, disruption of P2RY13, an adenosine diphosphate receptor expressed by microglia under basal conditions and absent in astrocytes, neurons, and neural stem cells, increases the proliferation of progenitor cells and formation of new neurons, which suggests additional mechanisms of homeostasis that regulate adult neurogenesis [35]. Other receptors involved in the regulation of adult neurogenesis, as mentioned above, include CX3CR1 (expressed mainly in microglia). CX3CR1^-/-^ mice present with altered synaptic morphology and impaired connectivity of mature newborn granular cells, in addition to changes in emotional behavior [119]. Furthermore, microglia modulate the activation of neural precursors following physical activity in adult mice, increasing the activity of these cells and revealing that CX3CL1–CX3CR1 signaling is essential for this modulation [120]. Disruption of CX3CL1–CX3CR1 signaling by gene ablation or pharmacological blockage impairs adult neurogenesis [36,37]. In subsequent work, Maggi and colleagues showed that CC3CL3^−/−^ mice have decreased neurogenesis compared to wild-type mice [36]. The same effect was found by Bachstetter and colleagues, who reported that CX3CR1 inhibition reduces neurogenesis in young mice [37]. These findings support the critical involvement of microglia in the generation of new neurons and, consequently, the modulation of behaviors related to new-born adult neurons.

All of these studies, which were published mainly in the last decade, show that microglia are intimately related to adult neurogenesis and regulation of proliferation, survival, and integration of these new neurons into existing circuitry. As the addition of new neurons is closely associated with homeostasis and cognitive processes, it seems reasonable to suggest that microglia play a role in brain homeostasis and learning and memory processes in adulthood.

## 5. Conclusions

Microglial cells play a critical role when the brain suffers injury. However, these cells are constantly responding to neuronal signals by extending and withdrawing their processes from around synapses. Though the CSFR1 inhibition studies suggest that the brain can function perfectly well without microglia, it seems likely that the behavioral studies may not be sensitive enough to detect the contribution of microglia to plasticity, adult neurogenesis, and cognitive processes.

Therefore, though microglial function remains an important target for therapeutic intervention, it is important to recognize that these interventions likely impact normal neuronal function and the maintenance of brain homeostasis, as well as cognition and behavior.

## Figures and Tables

**Table 1:** Overview of the evidence accumulated on the roles of microglia in the healthy adult brain.

Activity	Function	Action	Reference
Homeostasis and Cognition	Neural environment monitoring and response to damage	Microglia respond to molecular signals such as ATP.	[5,6]
		Microglial processes contact synapses, peri-synaptic astrocytes and synaptic clefts.	[7,8]
	Microglial morphology dynamics controlled by neurotransmission	Receptor expression for neurotransmitters, allowing response to synaptic activity and release of molecules (e.g., chemokines and cytokines).	[9,10,11,12]
		Regulation controlled by the levels of glutamate and Gamma aminobutyric acid (GABA) in addition to ATP.	[8,13,14]
	Neuronal modulation of microglial functions, ‘On’ mode	Increased levels of purines, such as ATP and UTP, induce microglial activation with migration to sites of damage and increased phagocytic activity.	[6,15]
		Increased levels of purines, cytokines, and glutamate act as ‘On’ signals, inducing microglial activation (pro-inflammatory or neuroprotective).	[16,17]
	Neuronal modulation of microglial functions, ‘Off’ mode	CD200, CX3CL1, and TREM2 deficit increases microglial activation, resulting in reduced synaptic plasticity and reduced phagocytic activity.	[18,19,20,21,22]
	Microglial signaling	Silencing transforming growth factor beta-1 (TGFβ1) signaling in microglia results in disturbed homeostasis.	[23]
		Deletion of TGF-β receptor type II (TGFβr2) causes microglial activation and upregulation of priming markers.	
		Microglial brain-derived neurotrophic factor (BDNF) signaling plays a crucial role in learning and memory-related synaptic plasticity through the tropomyosin-related kinase receptor B signaling pathway.	[24]
	Microglia in cognitive processes	Microglial replacement after depletion by colony stimulating factor 1 receptor (CSF1R) inhibition restores aging-altered neuronal gene expression, improving brain homeostasis and cognitive processes.	[25,26]
	Synaptic pruning	Microglia exhibit phagocytic behavior, engulfing synaptic elements in elderly animals with deficits in auditory function.	[27]
	Cell communication	Synaptic activity increases and the neuronal population synchronizes after microglial processes contact spines that are not detected when microglia are activated by lipopolysaccharide.	[28]
		Microvesicles released from microglia affect excitatory neurotransmission by stimulating the neuronal production of ceramide and sphingosine.	[29]
Neurogenesis	Enriched environment	Microglial activation increases adult neurogenesis induced by an enriched environment.	[30]
	Soluble factors	*In vitro* microglial depletion correlates with the loss of inducible neurogenesis and the microglial environment can rescue it.	[31]
		Cell culture media from BV2 immortalized microglia increases the proliferation of adult mouse-derived neural stem/progenitor cells.	[32]
	Contact	Unchallenged microglia from the subgranular zone quickly and exclusively phagocytose and clear the apoptotic neurons, maintaining the homeostasis of the neurogenic cascade.	[33]
	Molecular profile	Compared to microglia residing elsewhere in the hippocampus, microglial cells from the neurogenic zone in the dentate gyrus exhibit a unique RNA expression profile, responding exclusively to neurogenic factor Vascular endothelial growth factor (VEGF). Even reduced microglial number leads to a reduction of the number of new neuroblasts.	[34]
	Receptor modulation	The disruption of *P2ry13* increases proliferation of progenitor cells and the formation of new neurons, pointing to additional mechanisms of homeostasis control regulating adult neurogenesis.	[35]
		*CX3CR1* and *Cx3cl3* blockage reduces adult neurogenesis.	[36,37]
